# Correction: Necessity of two-dimensional visualization of validity in the nanomechanical mapping of atomic force microscopy for sulphur cross-linked rubber

**DOI:** 10.1039/d5ra90129d

**Published:** 2025-11-07

**Authors:** Takumi Ohashi, Tomoyuki Sato, Taichi Nakajima, Preeyanuch Junkong, Yuko Ikeda

**Affiliations:** a Graduate School of Science and Technology, Kyoto Institute of Technology Matsugasaki, Sakyo Kyoto 606-8585 Japan; b Center for Rubber Science and Technology, Kyoto Institute of Technology Matsugasaki, Sakyo Kyoto 606-8585 Japan; c Research Strategy Promotion Center, Kyoto Institute of Technology Matsugasaki, Sakyo Kyoto 606-8585 Japan; d Faculty of Molecular Chemistry and Engineering, Kyoto Institute of Technology Matsugasaki, Sakyo Kyoto 606-8585 Japan yuko@kit.ac.jp

## Abstract

Correction for ‘Necessity of two-dimensional visualization of validity in the nanomechanical mapping of atomic force microscopy for sulphur cross-linked rubber’ by Takumi Ohashi *et al.*, *RSC Adv.*, 2018, **8**, 32930–32941, https://doi.org/10.1039/C8RA06669H.

The ORCiD previously listed for Yuko Ikeda was incorrect. The correct ORCiD is: 0000-0003-2809-8911.

The Royal Society of Chemistry apologises for these errors and any consequent inconvenience to authors and readers.

